# A Case Report of Acquired ALK Fusion in ALK Wild‐Type Lung Adenocarcinoma Following Chemotherapy and a Literature Review Is Presented

**DOI:** 10.1002/rcr2.70501

**Published:** 2026-02-22

**Authors:** Wenqing Cheng, Ganling Qiao, Wenhua Zhang, Yifan Song, Xiangyi Zan

**Affiliations:** ^1^ The Second Hospital of Lanzhou University Lanzhou Gansu Province China

**Keywords:** acquired ALK fusion, lung adenocarcinoma, post‐chemotherapy

## Abstract

ALK fusion is a key driver mutation in non‐small cell lung cancer, typically present as a primary genetic alteration. This article reports a rare case of a patient who was initially ALK‐negative but subsequently developed an ALK fusion following chemotherapy. A 52‐year‐old female was diagnosed with advanced lung adenocarcinoma. Initial genetic testing showed wild‐type EGFR and ALK. She received first‐line platinum‐based doublet chemotherapy combined with a VEGF inhibitor and a PD‐L1 inhibitor, achieving a partial response. Upon disease progression, repeat genetic testing unexpectedly detected an ALK fusion. Treatment was then switched to the ALK inhibitor alectinib, and the patient again achieved a partial response. This case suggests that chemotherapy may enrich ALK fusion‐positive tumour cell clones through selective pressure. These findings highlight the clinical importance of repeated genetic testing after disease progression and provide new insights for post‐resistance treatment strategies.

## Graphical abstract

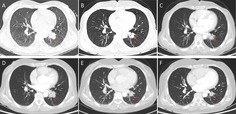

## 1 Introduction

Lung cancer remains the leading cause of cancer‐related mortality worldwide, with non‐small cell lung cancer (NSCLC) accounting for approximately 85% of cases. The treatment of advanced NSCLC has evolved from conventional chemotherapy to driver gene‐based individualised targeted therapy. Agents directed against various targets such as EGFR and ALK have become first‐line standard regimens, significantly prolonging patient survival and transitioning lung cancer into an era of chronic disease management [1]. ALK fusion is identified in approximately 3%–7% of NSCLC cases, representing a relatively uncommon driver alteration. In contrast to EGFR mutations, ALK fusions are more frequently observed in younger patients (typically under 55 years of age), never‐ or light‐smokers with adenocarcinoma, particularly those containing signet‐ring cell components [2]. Although the vast majority of ALK fusions are primary events, acquired ALK fusions are exceedingly rare. They have been occasionally reported following resistance to EGFR‐TKIs [3], but cases reported after chemotherapy are even more uncommon. This report presents a rare case of ALK fusion emerging after chemotherapy and discusses its potential mechanisms and clinical implications.

## 2 Case Report

**FIGURE 1:**
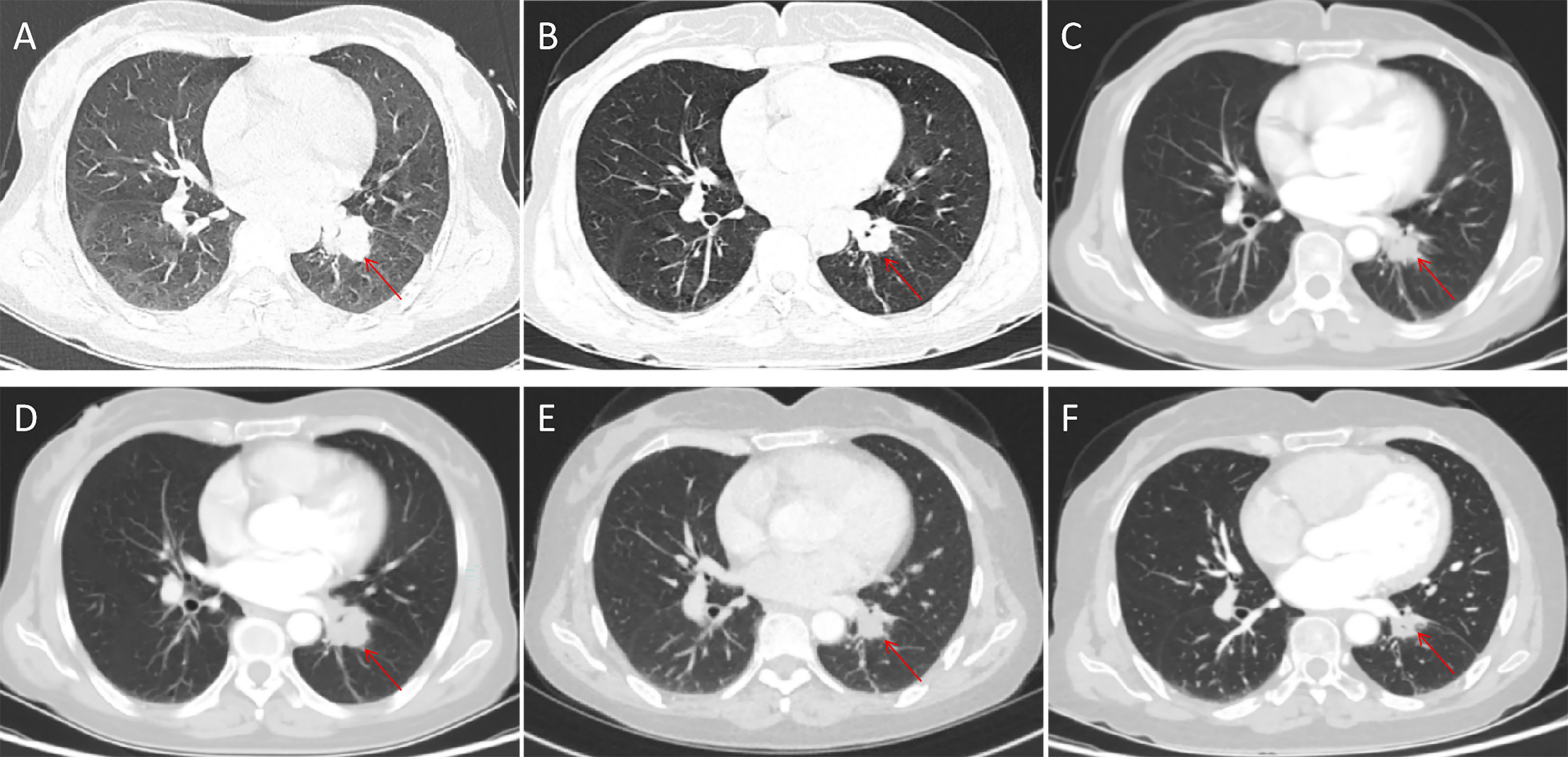
(A) Initial diagnosis of lung adenocarcinoma; (B) After 3 months of chemotherapy; (C) After 1 year of chemotherapy; (D) Disease progression; (E) After 2 months of targeted therapy following progression; (F) After 1 year of targeted therapy following progression.

A 52‐year‐old non‐smoking female presented in March 2022 with a six‐month history of cough, expectoration, and left‐sided chest and back pain. Imaging studies identified a 2.2 × 1.8 cm mass in the left lower lung lobe (Figure 1A) accompanied by enlarged hilar and mediastinal lymph nodes, bilateral adnexal masses and multiple systemic bone metastases. Bronchoscopic biopsy of a lesion in the left lower lobe (specimen diameter: 0.4 cm) confirmed the diagnosis of lung adenocarcinoma. Immunohistochemical staining showed positivity for CKpt^+^, TTF‐1^+^, and CK7^+^, weak positivity for NapsinA, and negativity for CK5/6^−^, p40^−^, Syn^−^ and CgA^−^. CD56 showed focal positivity, and the Ki‐67 index was approximately 15%. Genetic testing revealed wild‐type EGFR and no ALK fusion. The patient was diagnosed with left lung adenocarcinoma (stage cT1bN3M1c IVB). The patient began treatment in April 2022 with eight cycles of pemetrexed, cisplatin and bevacizumab. In March 2023, the treatment regimen was changed to three cycles of liposomal paclitaxel, cisplatin, bevacizumab and pembrolizumab, resulting in a partial response (Figure 1B,C). This was followed by six cycles of maintenance therapy with bevacizumab and pembrolizumab. Three months after treatment discontinuation, a follow‐up examination showed an increase in lesion size to 2.3 × 2.6 cm (Figure 1D), indicating disease progression (PD). Repeat biopsy and genetic testing revealed an ALK fusion. The patient was started on alectinib (600 mg twice daily). After 2 months of targeted therapy, re‐evaluation showed a reduction in lesion size to 2.0 × 1.6 cm (Figure 1E), again assessed as PR. The patient has now been on alectinib for 14 months. Recent imaging demonstrates further shrinkage of the lesion to 1.5 × 1.2 cm (Figure 1F), as well as improvement in bone metastases and adnexal lesions.

All ALK testing (both at baseline and upon progression) in this study was performed in the Department of Pathology of Lanzhou University Second Hospital. The widely validated Ventana ALK (D5F3) CDx Assay immunohistochemistry (IHC) method was uniformly applied. Staining was interpreted by an experienced pathologist in strict accordance with CAP/AMP guidelines, with positivity defined as strong, complete membranous staining in ≥ 1% of tumour cells. Previous studies have confirmed that this detection system has a sensitivity of > 95% for ALK rearrangements in lung adenocarcinoma [4]. The use of the same IHC platform, reagents and interpretation criteria for both baseline and follow‐up testing ensured methodological consistency.

## 3 Discussion

This case report describes a patient with advanced lung adenocarcinoma who, following progression on platinum‐based chemotherapy and immunotherapy, was found to have an acquired ALK fusion during secondary genetic testing and ultimately benefited from ALK‐TKI therapy. This case provides important insights into understanding tumour resistance mechanisms and clinical decision‐making.

ALK fusions are typically considered primary drivers in NSCLC [2]. In this case, initial ALK testing was negative, yet an ALK fusion was newly detected after chemotherapy. Such ‘acquired’ rearrangements are extremely rare. Existing reports indicate that acquired ALK fusions predominantly emerge after EGFR‐TKI resistance, often via bypass activation mechanisms [3]. This case suggests that conventional chemotherapy can also exert powerful evolutionary pressure, driving tumour genomic rearrangements.

Regarding the mechanism, we favour the clonal selection hypothesis [5]. This suggests that a small subclone carrying the ALK fusion, inherently resistant to chemotherapy, may have existed in the initial tumour but remained undetected due to its abundance falling below the detection limit. Platinum‐based chemotherapy eliminated the dominant sensitive clones, creating space for this resistant clone to expand and ultimately become the predominant population. Another possibility—chemotherapy directly inducing genomic instability leading to ALK rearrangement [5]—remains theoretically plausible but lacks evidence and is considered less likely.

The core clinical value of this case lies in confirming the necessity of repeated genetic testing during disease progression. Regardless of prior treatment regimens, once disease progresses, re‐biopsy should be performed to identify new targetable mechanisms, which is crucial for securing precision treatment opportunities for patients. Upon detection of acquired ALK fusions, immediate conversion to ALK‐TKI targeted therapy is warranted. This patient achieved rapid partial remission after alectinib treatment, fully demonstrating the exceptional efficacy of late‐line precision targeted therapy.

This study also has several limitations. First, as a single‐case report, it cannot estimate the overall incidence of such acquired ALK fusions. Second, due to the limited baseline biopsy specimen (small volume), we were unable to retrospectively perform ultra‐deep sequencing to verify whether low‐abundance ALK‐fusion clones existed in the initial tissue. Thus, direct molecular evidence supporting the clonal selection hypothesis remains unavailable. Third, the small baseline biopsy itself carries a risk of false‐negative ALK IHC results due to tumour heterogeneity and sampling bias. Given these specimen‐ and evidence‐related constraints, the exact causal relationship between chemotherapy and the detection of the ALK fusion remains unclear. The previously mentioned notion that ‘chemotherapy might induce gene rearrangement’ remains only a mechanistic hypothesis; an explanation more consistent with current tumour evolution theory is that chemotherapy exerted clonal selection pressure, promoting the expansion of pre‐existing ALK‐fusion‐positive subclones into the dominant population. The causal relationship between chemotherapy and the emergence of the ALK fusion requires validation in future prospective studies using paired pre‐ and post‐treatment specimens and deep‐sequencing technologies.

## Author Contributions

**Wenqing Cheng:** data curation, investigation, writing – original draft. **Ganling Qiao, Wenhua Zhang, Yifan Song:** investigation, visualisation, writing – review and editing. **Xiangyi Zan:** conceptualisation, supervision, project administration, writing – review and editing.

## Funding

This work was supported by the Natural Science Foundation of Gansu Province (22JR5RA993), Lanzhou University Second Hospital Cuiying Program (CY2023‐MS‐B11), Gansu Provincial Drug Regulatory Science Research Project (2023GSMPA019).

## Consent

The authors declare that written informed consent was obtained for the publication of this manuscript and accompanying images using the consent form provided by the Journal.

## Conflicts of Interest

The authors declare no conflicts of interest.

## Data Availability

The data that support the findings of this study are available on request from the corresponding author. The data are not publicly available due to privacy or ethical restrictions.
